# Bond strength of orthodontic brackets to CAD/CAM ceramics following various surface treatments

**DOI:** 10.1007/s00784-026-06784-0

**Published:** 2026-02-21

**Authors:** Gulben COLAK, Elif ALBAYRAK, Muhittin UGURLU

**Affiliations:** 1 Department of Restorative, Faculty of Dentistry, Süleyman Demirel University, Isparta, Turkey; 2 Department of Orthodontics, Faculty of Dentistry, Süleyman Demirel University, Isparta, Turkey

**Keywords:** Shear bond strength, Orthodontic brackets, CAD/CAM ceramics, Surface treatment, Lithium disilicate, Feldspathic porcelain

## Abstract

**Objectives:**

This study evaluated the shear bond strength (SBS) of two types of orthodontic brackets (ceramic and metal) bonded to two CAD/CAM ceramic materials (feldspathic ceramic and lithium disilicate glass-ceramic), each subjected to different surface treatments, and assessed adhesive remnant index (ARI) scores.

**Materials and methods:**

Sixty-four ceramic discs (1 mm thick; *n* = 32 per material) were prepared. Surface pretreatments were alumina air abrasion followed by phosphoric acid etching or hydrofluoric acid etching. Each subgroup was bonded with metal or ceramic brackets using a light-cured adhesive. SBS was measured with a universal testing machine, and ARI was assessed under a stereomicroscope. Three-way ANOVA tested the effects of ceramic type, bracket type, and surface treatment (α = 0.05), and chi-square tests compared ARI distributions.

**Results:**

The results demonstrated that the ceramic type significantly influenced SBS (*p* = 0.004). Lithium disilicate glass-ceramic exhibited higher SBS values than feldspathic ceramic. Bracket type also had a significant effect on SBS, regardless of ceramic type or surface treatment (*p* = 0.001). However, the applied surface treatments did not produce a significant difference in SBS (*p* = 0.546); phosphoric acid etching after alumina air abrasion yielded comparable results to hydrofluoric acid etching. ARI scores did not differ significantly among groups, and debonded surfaces showed no gross ceramic damage under stereomicroscopy.

**Conclusions:**

Ceramic type and bracket type significantly affected SBS, whereas surface pretreatment did not under the tested conditions.

**Clinical relevance:**

Balancing adequate bracket retention with safe debonding is essential for ceramic restorations. Comparable SBS between the two pretreatments and the absence of visible ceramic damage support conservative, clinically oriented conditioning protocols.

## Introduction

Among adult patients, orthodontic treatment is becoming more and more common; many of them already have indirect ceramic restorations [1]. Bonding brackets to these surfaces presents a therapeutic challenge since ceramic materials have different mechanical properties and chemical compositions. The adhesive contact has to withstand masticatory and orthodontic pressures without compromising the integrity of the restoration or leading to bracket failure [2, 3]. With the advancement of digital dentistry, CAD/CAM-fabricated ceramics have become popular due to their aesthetic appeal, biocompatibility, and durability [4, 5]. Common materials include feldspathic ceramics and lithium disilicate glass ceramics, which differ significantly in structure, translucency, and surface reactivity [6, 7]. Feldspathic ceramics comprise feldspathic crystal particles in a glassy matrix, while lithium disilicate ceramics offer superior translucency, aesthetics, and strong bonding to tooth surfaces [8–10].

The dissolution of acid-sensitive silica in glass-ceramics can influence bond strength, improving micromechanical/chemical adhesion to ceramic surfaces [11, 12]. To increase bracket bonding to ceramics, several surface treatments—including mechanical and chemical ones—have been used [11, 13]. Although hydrofluoric acid has been demonstrated to boost adhesion, its potential to injure soft tissues calls for careful use by clinicians [14–17]. Another etching choice is phosphoric acid, which reduces bond strength relative to hydrofluoric acid. Further mechanical roughening is often required to attain adequate bonding to ceramic restorations [18, 19]. Orthodontic brackets, available in metal and ceramic varieties, are used to align and straighten teeth. Metal brackets are typically stainless steel. Ceramic brackets offer superior aesthetics and color stability and are nickel-free; however, their hardness may complicate removal and increase the risk of enamel damage [20, 21].

Consequently, this study intends to assess the shear bond strength (SBS) of orthodontic ceramic and metal brackets bonded to two different CAD/CAM ceramic blocks, utilizing two common surface pretreatment protocols. It will also evaluate the adhesive remnant index (ARI) following debonding. In line with this objective, the following null hypotheses were tested:

1. The type of ceramic block does not affect the SBS of orthodontic metal and ceramic brackets.
2. The bracket type does not influence SBS, and.
3. Different surface treatments do not alter the SBS of orthodontic brackets when used with distinct CAD/CAM ceramics.

## Materials and methods

### Sample preparation

Two kinds of CAD/CAM ceramic blocks were tested: lithium disilicate glass ceramic (CEREC Tessera; Dentsply Sirona) and feldspathic ceramic (CEREC Blocs C; Dentsply Sirona) (Table 1). A total of 64 rectangular-shaped specimens (32 per material) were prepared from each block using a precision cutting machine, 12.4 × 14.5 mm in dimension and 1 mm in thickness.

**Table 1:** CAD-CAM ceramic materials used in the study

Material	Composition	Manufacturer	Lot no
Lithium disilicate glass ceramic (CEREC Tessera)	SiO_2,_ Li_2_O, K_2_O, P_2_O_5_, ZrO_2_, ZnO, Al_2_O_3_, MgO	Dentsply (Milford, USA)	16,015,103
Feldspathic ceramic (CEREC Blocs C)	SiO_2,_ Al_2_O_3_, Na_2_O, K_2_O, CaO, TiO_2_	Dentsply (Milford, USA)	6,484,674

### Surface treatment

Each ceramic group was further divided into two subgroups (*n* = 16) according to surface treatment:

Group A (air abrasion + phosphoric acid): Air abrasion was performed using 50 μm aluminum oxide (Al_2_O_3_) at 2 bar for 20 s, followed by 37% phosphoric acid (Transbond XT Etching Gel; 3 M Unitek) for 15 s. The distance between the air-abrasion tip and the specimen surface was set at 3 mm, and a nozzle tip with a diameter of 0.6 mm was used.

Group B (hydrofluoric acid): 10% hydrofluoric acid (HF) was applied for 20 s. All treated surfaces were then rinsed with water and dried with oil-free air.

### Bracket bonding procedure

Each subgroup was bonded with 8 metal and 8 ceramic upper incisor brackets (MBT, 0.022-inch slot). The ceramic brackets used in this study were composed of alumina ceramic, an oxide-based, glass-free polycrystalline material predominantly consisting of aluminum oxide (Al₂O₃) and characterized by strong ionic-covalent bonding and high chemical stability.

The bonding protocol followed manufacturer instructions using a light-cured adhesive (Transbond XT; 3 M Unitek) and LED curing unit (Valo; Ultradent) at 1000 mW/cm². Excess resin was removed with a periodontal probe. Curing was performed for 40 s, maintaining the light tip 1–2 mm from the bracket.

### Storage and shear bond strength testing

**Fig. 1:**
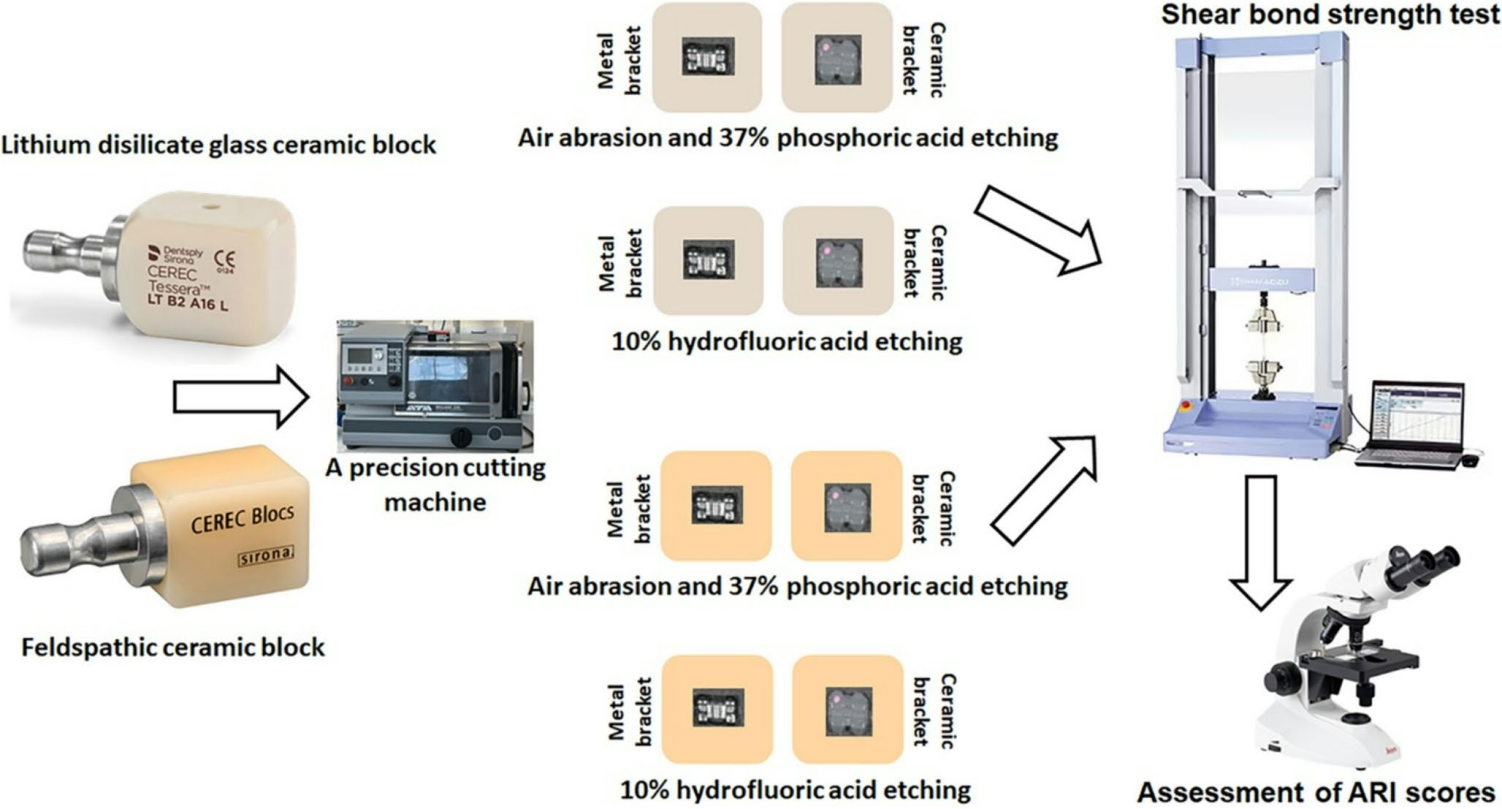
Schematic illustration of the experimental design

Specimens were stored in distilled water at 37 °C for 24 h before testing. SBS was tested with a universal testing machine (Autograph AGS-X; Shimadzu, Japan) by applying a sideways force on the disc surface at a speed of 1 mm/min. The force (N) was divided by the bracket base area (mm²) to calculate SBS in MPa (Fig. 1. Schematic illustration of the experimental design).

### Adhesive remnant index (ARI)

Following debonding, residual adhesive on each specimen was assessed under a stereomicroscope (S4E; Leica Microsystems) at 20× magnification. The ARI scores were classified as [22]:

0 = no adhesive remaining;

1 = < 50% adhesive remaining;

2 = > 50% adhesive remaining;

3 = 100% adhesive remaining.

### Statistical analysis

Data were analyzed using SPSS version 29.0 (IBM, Chicago, IL, USA). The Kolmogorov–Smirnov test confirmed normal distribution. A three-way ANOVA was used to assess the effects of ceramic type, bracket type, and surface treatment. ARI scores were compared using the chi-square test. Statistical significance was set at *p* < 0.05.

This in vitro study did not involve human or animal subjects and therefore did not require ethical approval.

## Results

**Fig. 2:**
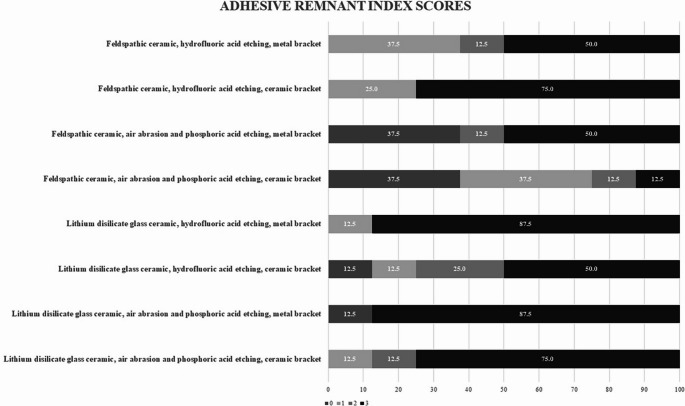
The frequencies of adhesive remnant index (ARI) scores (%) observed using light microscopy

**Fig. 3:**
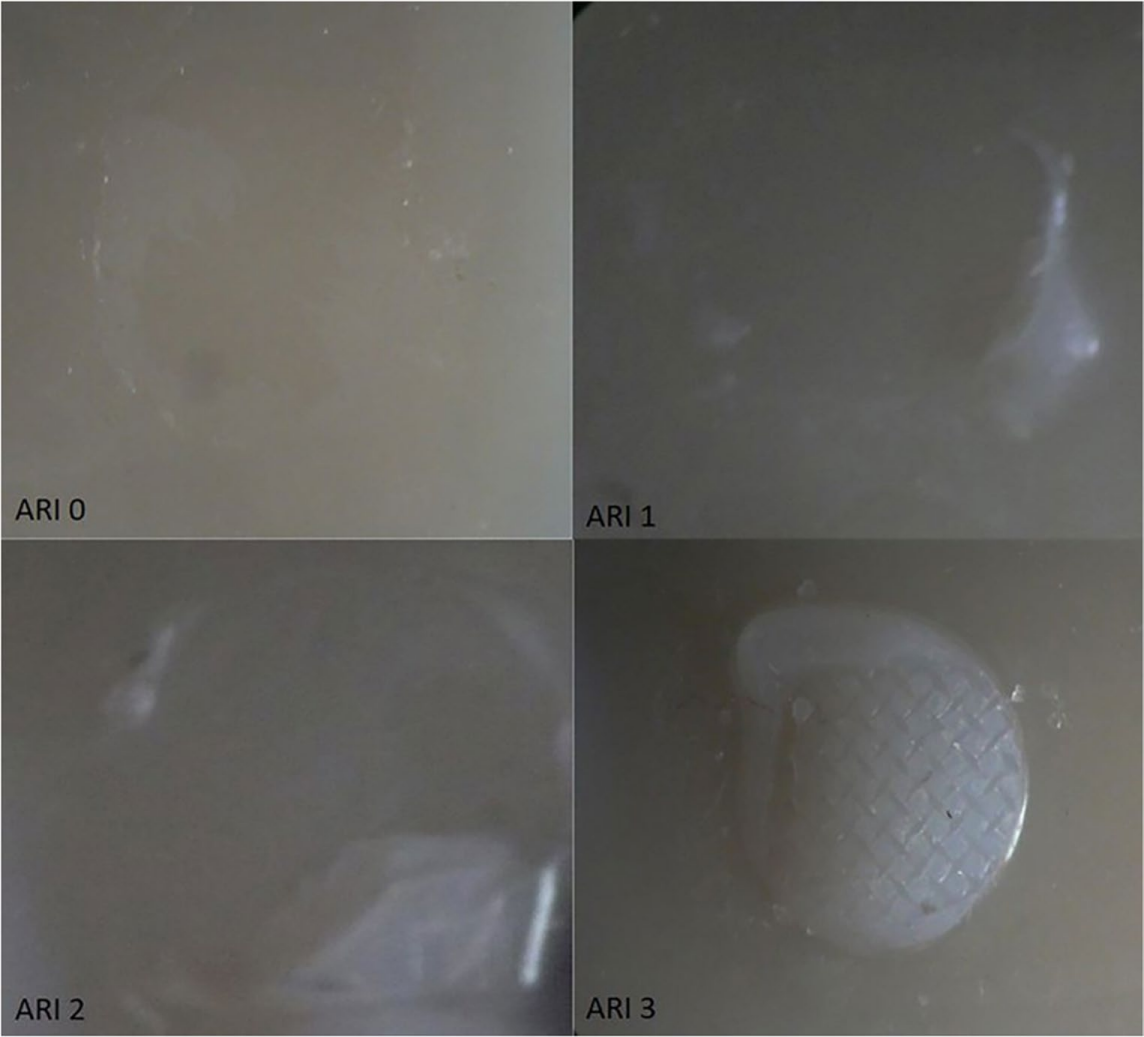
Stereomicroscope images (20×) of debonded ceramic surfaces illustrating ARI categories: (A) **ARI 0** — no adhesive remaining on the ceramic; (B) **ARI 1** — <50% adhesive remaining; (C) **ARI 2** — >50% adhesive remaining; (D) **ARI 3** — 100% adhesive remaining. *ARI*,* Adhesive Remnant Index*

Table 2 shows the results of the three-way ANOVA. The effect sizes were small-to-moderate (partial η²: ceramic type = 0.142; bracket type = 0.179). Table 3 lists the means and standard deviations of the SBS values for the experimental groups. Figure 2 presents the distribution of ARI scores. ARI scores did not differ significantly across ceramic type, bracket type, or surface pretreatment (chi-square tests, *p* > 0.05). During debonding, the bond-failure location varied across groups; no single interface (bracket–adhesive or ceramic–adhesive) predominated. Figure 3 displays light-microscopy images of debonded ceramic surfaces, illustrating micro-retentive features in line with pretreatments and no gross surface cracks or defects. Ceramic type had a statistically significant effect on SBS (*p* = 0.004), with lithium disilicate glass ceramics showing higher values. Surface pretreatment did not significantly influence SBS (*p* = 0.546), with all methods yielding similar results. Bracket type also significantly affected SBS (*p* = 0.001), with ceramic brackets performing better than metal brackets. However, no significant differences were observed for the interaction of all three factors (*p* = 0.960).

**Table 2:** The three-way ANOVA test results for the shear bond strength test

Source	Type III sum of squares	df	Mean square	F	Sig.	Partial Eta squared
Corrected Model	107,369^a^	7	15,338	3,130	0,007	0,281
Intercept	6706,996	1	6706,996	1368,832	0,000	0,961
Ceramic type	45,276	1	45,276	9,240	0,004^*^	0,142
Surface pretreatment	1,806	1	1,806	0,369	0,546	0,007
Bracket type	59,850	1	59,850	12,215	0,001^*^	0,179
Ceramic type* Surface pretreatment	0,232	1	0,232	0,047	0,829	0,001
Ceramic type* Bracket type	0,003	1	0,003	0,001	0,979	0,000
Surface pretreatment* Bracket type	0,190	1	0,190	0,039	0,844	0,001
Ceramic type* Surface pretreatment* Bracket type	0,012	1	0,012	0,003	0,960	0,000
Error	274,388	56	4,900			
Total	7088,753	64				
Corrected Total	381,758	63				

**Table 3:** The mean shear bond strengths (MPa ± SD) of the different experimental groups

Ceramic type	Surface pretreatment	Metal bracket	Ceramic bracket
Lithium disilicate glass ceramic (CEREC Tessera)	Air abrasion and 37% phosphoric acid etching	9.84 ± 2.01	11.87 ± 2.52
	10% hydrofluoric acid etching	10.37 ± 2.06	12.24 ± 2.75
Feldspathic ceramic (CEREC Blocs C)	Air abrasion and 37% phosphoric acid etching	8.26 ± 1.82	10.32 ± 2.18
	10% hydrofluoric acid etching	8.61 ± 1.84	10.40 ± 2.35

## Discussion

With technological advances, CAD/CAM applications have become increasingly popular for restoring teeth with substantial material loss. However, orthodontic treatments involving ceramic restorations remain challenging, as brackets bonded to ceramic surfaces tend to fail more often than those bonded to enamel [23, 24]. Bonding outcomes are influenced by several factors, including porcelain type, surface characteristics, bracket material, bonding agent, curing method, and clinician experience. Achieving adequate bond strength while ensuring safe debonding is essential to prevent damage to restored teeth [11, 25].

This study evaluated the shear bond strength (SBS) of two types of orthodontic brackets (ceramic and metal) bonded to two CAD/CAM ceramic materials (feldspathic ceramic and lithium disilicate glass-ceramic), each subjected to different surface treatments (37% phosphoric acid etching after alumina air abrasion vs. 10% hydrofluoric acid etching). In addition, adhesive remnants on debonded ceramic surfaces were assessed under a stereomicroscope using the ARI.

The results demonstrated that the ceramic type significantly influenced SBS. Lithium disilicate glass-ceramic exhibited higher SBS values than feldspathic ceramic. Pulido et al. reported that the chemical composition of ceramics strongly affects the efficacy of surface treatments, as identical treatments can yield distinct surface morphologies depending on the ceramic material [26]. In line with recent evidence, lithium disilicate demonstrates superior SBS relative to feldspathic ceramics when assessed under comparable, clinically relevant conditioning protocols [27]. Feldspathic ceramics differ in chemical composition and component percentage compared to lithium disilicate ceramics [8]. Consequently, these microstructural differences lead to distinct responses to hydrofluoric acid etching. Feldspathic ceramics exhibit micropores, channels, and irregularly distributed particles, while lithium disilicate ceramics display elongated or bean-shaped crystals due to silica matrix dissolution [28]. Consistent with previous studies, the present findings confirm that ceramic type primarily determines surface roughness and, therefore, SBS [27–29]. Accordingly, the first hypothesis of this study—that the ceramic type does not influence the SBS of ceramic brackets and metal brackets—was rejected.

Bracket type also had a significant effect on SBS, regardless of ceramic type or surface treatment. In agreement with Elsaka SE (2016), Babaee Hemmati Y et al. (2022), and Haralur SB et al. (2023), ceramic brackets showed higher SBS than metal brackets [30–32]. This may be attributed to the ceramic bracket base design, which promotes micromechanical interlocking with orthodontic adhesives due to polycrystalline alumina’s rough surface containing randomly aligned crystals and spherical glass particles [31]. Thus, the second hypothesis of this study—that bracket type would not affect SBS—was rejected.

Bonding orthodontic brackets to ceramic surfaces is inherently difficult, and various surface-conditioning techniques have been proposed [13, 31, 33]. These techniques are commonly classified as chemical, mechanical, or combined [33]. Mechanical approaches such as alumina air abrasion have been reported to increase bond strength in some studies [13, 33, 34], and chemical etching—most frequently with 10% hydrofluoric acid (HF)—can also improve adhesion to ceramics [23, 31, 32, 34]. However, reported effects vary with ceramic composition and protocol, and several reports indicate comparable outcomes between HF etching and combined abrasion-plus-phosphoric-acid approaches [13, 18]. In this study, the applied surface treatments did not produce a significant difference in SBS (*p* = 0.546); air abrasion followed by phosphoric acid yielded values comparable to HF etching. Therefore, the third null hypothesis—that surface treatments do not affect SBS—was accepted.

In this study, alumina air abrasion was applied to the ceramic surface using conservative parameters (50 μm Al₂O₃, 2 bar, 20 s) to achieve controlled microroughening while minimizing the risk of surface damage. The subsequent application of 37% phosphoric acid was used not to aggressively etch the glass-ceramic, but as a cleaning and surface-wettability–enhancing step to remove abrasive residues and facilitate silane/adhesive interaction [15, 32, 35]. Evaluating the present results, the air abrasion **+** phosphoric acid approach yielded SBS values comparable to hydrofluoric acid etching (*p* = 0.546). These findings support that combining a mechanical roughening step with a phosphoric-acid cleaning/priming step can provide reliable bonding on CAD/CAM ceramics.

In clinical practice, the clinician’s priority is to remove brackets without compromising the underlying ceramic restoration. Ideally, debonding occurs at the bracket-adhesive interface, leaving the adhesive on the ceramic. This protects the restoration but requires more chairside cleaning. Debonding at the ceramic-adhesive interface leaves less adhesive to be removed, but the risk of damage to the ceramic surface is higher. ARI scoring determines the location of failure by showing how much adhesive remains on the ceramic after debonding. In this study, ARI distributions did not reflect bond strength: despite differences in ceramic and bracket type, no significant association between SBS and ARI was observed (chi-square tests, *p* > 0.05). While some studies have observed that groups with higher SBS tended to exhibit higher ARI scores [28, 32, 36], a contemporary systematic review including 32 studies found no consistent SBS-ARI relationship [37]. This is thought to be because the ARI value is influenced by many factors (adhesive chemistry, bracket base design, ceramic composition, and surface pretreatment) [38, 39]. In the present study, the two pretreatments tested (air abrasion + phosphoric acid vs. hydrofluoric acid) provided comparable SBS, and debonding did not occur at a single interface; no ceramic damage was observed by stereomicroscopic examination.

Reynolds reported that a shear bond strength of approximately 6–8 MPa represents the minimum requirement for effective clinical orthodontic bracket retention [40]. In contrast, Ozden et al. indicated that SBS values exceeding 13 MPa may predispose ceramic surfaces to fracture during bracket debonding [41]. In the present study, the mean SBS values ranged from 8.26 to 12.24 MPa across all experimental groups. These values exceeded the minimum threshold required for clinical retention while remaining below the range associated with an increased risk of ceramic damage. Therefore, the bond strength values obtained in this study can be considered clinically acceptable and safe for orthodontic bonding to CAD/CAM ceramic surfaces.

This study has several limitations. Only two surface treatment protocols and two ceramic materials were evaluated under static in vitro conditions. A further limitation of the present study is that the specimens were stored in water for only 24 h before shear bond strength testing, and no thermal cycling protocol was applied. Thermal cycling is known to simulate oral temperature fluctuations and may adversely affect the durability of the adhesive interface over time. Therefore, future studies should (i) assess long-term bond durability using standardized thermal cycling aging protocols, (ii) include non–silica-based ceramics, such as 5Y-TZP zirconia, using zirconia-appropriate bonding protocols, and (iii) compare additional surface treatments and evaluate their clinical performance in patients with CAD/CAM restorations. Additionally, SEM-based surface characterization before and after surface pretreatment would provide further insight into surface morphology changes. In the present study, hydrofluoric acid was used as a surface treatment for ceramic surface roughening. Hydrofluoric acid is a highly aggressive agent and may cause severe damage to soft tissues if accidental contact occurs in the oral environment. Therefore, when applied clinically, HF acid etching should be performed under strict isolation, preferably using a rubber dam, and contact with surrounding soft tissues should be minimized.

## Conclusion

Within the limitations of this in vitro study, lithium disilicate exhibited higher SBS than feldspathic ceramics. Ceramic brackets outperformed metal brackets irrespective of pretreatment. Surface pretreatment (air abrasion + phosphoric acid vs. hydrofluoric acid) did not significantly affect SBS. Clinically, the combination of lithium disilicate ceramics and ceramic brackets may provide superior bonding performance, potentially reducing bracket failure in patients with CAD/CAM restorations. When orthodontic treatment is planned following prosthetic rehabilitation, particularly in patients with high esthetic demands requiring ceramic brackets, selecting lithium disilicate as the restorative material may contribute to improved orthodontic treatment success by supporting more reliable bracket bonding.

## Data Availability

Data is provided within the manuscript. Further details are available from the corresponding author upon reasonable request.
